# Simplatab: An Automated Machine Learning Framework for Radiomics-Based Bi-Parametric MRI Detection of Clinically Significant Prostate Cancer

**DOI:** 10.3390/bioengineering12030242

**Published:** 2025-02-26

**Authors:** Dimitrios I. Zaridis, Vasileios C. Pezoulas, Eugenia Mylona, Charalampos N. Kalantzopoulos, Nikolaos S. Tachos, Nikos Tsiknakis, George K. Matsopoulos, Daniele Regge, Nikolaos Papanikolaou, Manolis Tsiknakis, Kostas Marias, Dimitrios I. Fotiadis

**Affiliations:** 1 Biomedical Research Institute, FORTH, GR 45110 Ioannina, Greece; dimizaridis@mail.ntua.gr (D.I.Z.); mylona.eugenia@gmail.com (E.M.); xkalantzopoulos@gmail.com (C.N.K.); ntachos@gmail.com (N.S.T.); 2 Unit of Medical Technology Intelligent Information Systems, University of Ioannina, GR 45110 Ioannina, Greece; bpezoulas@gmail.com; 3 Biomedical Engineering Laboratory, School of Electrical and Computer Engineering, National Technical University of Athens, GR 15780 Athens, Greece; gmatsopoulos@biomed.ntua.gr; 4 Institute of Computer Science, FORTH, GR 70013 Heraklion, Greece; tsiknakisn@ics.forth.gr; 5 Department of Radiology, Candiolo Cancer Institute, FPO-Istituto di Ricovero e Cura a Carattere Scientifico, Strada Provinciale 142 Km 3.95, IT 10060 Candiolo, Turin, Italy; daniele.regge@ircc.it; 6 Computational Clinical Imaging Group, Champalimaud Foundation, PT 1400-038 Lisboa, Portugal; nickolas.papanikolaou@research.fchampalimaud.org; 7 Computational Biomedicine Laboratory, Institute of Computer Science, FORTH, GR 70013 Heraklion, Greece; tsiknaki@ics.forth.gr (M.T.); kmarias@ics.forth.gr (K.M.); 8 Department of Electrical and Computer Engineering, Hellenic Mediterranean University, GR 71004 Heraklion, Greece

**Keywords:** prostate cancer, radiomics, MRI, artificial intelligence, automated machine learning framework, AutoML, open source

## Abstract

Background: Prostate cancer (PCa) diagnosis using MRI is often challenged by lesion variability. Methods: This study introduces Simplatab, an open-source automated machine learning (AutoML) framework designed for, but not limited to, automating the entire machine Learning pipeline to facilitate the detection of clinically significant prostate cancer (csPCa) using radiomics features. Unlike existing AutoML tools such as Auto-WEKA, Auto-Sklearn, ML-Plan, ATM, Google AutoML, and TPOT, Simplatab offers a comprehensive, user-friendly framework that integrates data bias detection, feature selection, model training with hyperparameter optimization, explainable AI (XAI) analysis, and post-training model vulnerabilities detection. Simplatab requires no coding expertise, provides detailed performance reports, and includes robust data bias detection, making it particularly suitable for clinical applications. Results: Evaluated on a large pan-European cohort of 4816 patients from 12 clinical centers, Simplatab supports multiple machine learning algorithms. The most notable features that differentiate Simplatab include ease of use, a user interface accessible to those with no coding experience, comprehensive reporting, XAI integration, and thorough bias assessment, all provided in a human-understandable format. Conclusions: Our findings indicate that Simplatab can significantly enhance the usability, accountability, and explainability of machine learning in clinical settings, thereby increasing trust and accessibility for AI non-experts.

## 1. Introduction

Prostate cancer (PCa) is a common cancer among men, a fact which highlights the critical need for timely diagnosis and staging to achieve effective treatment [1]. Despite the established diagnostic accuracy of MRI [2,3,4], the presence of lesions characterized by atypical or subtle characteristics, such as those often found in the peripheral zone, render visual assessment of the disease particularly challenging, increasing the chance of over- and under-diagnosis [5].

Artificial intelligence (AI) has made notable advancements in the domains of radiology and precision medicine, contributing to the emergence of radiomics as a promising avenue for supporting disease diagnosis [6,7,8,9,10,11,12]. Radiomics leverages the advantage of utilizing medical images for the generation and the exploration of non-invasive quantitative biomarkers related to tumor heterogeneity and biological characteristics. As opposed to relying on sampled data, radiomics harnesses the rich information embedded within these images with the aim of objectively and quantitatively describing and comprehensively analyzing the tumoral patterns otherwise invisible to the human eye [13].

A radiomics analysis involves several steps, such as the delineation of the region of interest (ROI) followed by the extraction of radiomic features [14], dimensionality reduction and feature selection, and finally model development and evaluation. Most commonly, the extracted features are formulated through mathematical equations and are known as “hand-crafted” features. These can be clustered into three main groups: (1) shape features, which assess morphological attributes, such as the elongation and the size of the ROI; (2) first-order (intensity-based) statistical features, which characterize the distribution of voxel intensities within the ROI, without consideration of their spatial relationships, and include measures such as mean, median, and standard deviation; and (3) second-order (texture-based) statistical features, which describe the statistical associations among the contrasting values of different voxels with the aim of quantifying intra-tumoral heterogeneity. It is also common to extract features not only from the original images but also after applying mathematical transformations to these images, such as Fourier or wavelet transforms, and include measures such as energy and entropy (higher-order features).

Doubtlessly, the feature extraction process results in a vast amount of radiomic features that need to be mined for both hypothesis generation and testing. With hundreds, or even thousands, of radiomic features originating from medical images, appropriate feature selection and model development become crucial [15], as redundancy and multicollinearity among the variables can have a detrimental impact on machine learning (ML) models, leading to misleading outcomes, overfitting, and reduced interpretability [16].

### Related Work

The emergence of big data technologies has increased the demand for fast, reliable, and robust machine learning (ML) pipelines. To meet this need, various automated machine learning (AutoML) frameworks have been developed to streamline the experimentation process for data scientists. Most of them are based on well-known libraries such as scikit-learn [17] and WEKA [18], and they have extended the notion of a streamlined ML process by automating the hyperparameters optimization, feature selection, and preprocessing of the data. For instance, Auto-WEKA [19], which is an AutoML tool integrated with the WEKA software (v3.8.6), is known for its widely used suite of ML algorithms and tools. It uses Bayesian optimization to automate the selection of algorithms and hyperparameters, making it easier for users to find the best model for their datasets without extensive manual experimentation. This tool is notable for its ability to explore a vast search space of algorithm configurations efficiently, leveraging the robust infrastructure of WEKA. Furthermore, Auto-Sklearn (v0.15.0) [20] is another popular library which is built on the widely used Python’s scikit-learn library. The library enhances scikit-learn’s capabilities by automating the process of model selection and hyperparameter tuning using Bayesian optimization and meta-learning. Auto-Sklearn also supports the construction of ensembles and is linked with scikit-learn’s extensive suite of ML algorithms, making it a powerful tool for users looking to streamline their model-building process while achieving high performance. The ML-Plan (Machine Learning Plan) (v0.2.5) [21] is another AutoML tool which utilizes hierarchical planning techniques to explore the space of possible machine learning pipelines. It constructs pipelines by combining different preprocessing steps, feature selection methods, and learning algorithms. ML-Plan’s planning-based approach allows an effective search through complex pipeline configurations and is particularly useful for users who deal with raw data that require advanced data processing workflows. Auto Tune Models (ATM) (v0.2.2) [22] is another suite of tools and techniques which aim to automate the hyperparameter tuning process for ML modeling. This suite of tools includes various libraries and frameworks that utilize methods such as grid search, random search, and more advanced techniques like Bayesian optimization and genetic algorithms to find the optimal hyperparameters for a given model. ATM is designed to enhance the performance of machine learning models by systematically exploring and tuning the parameters that control the learning process. Google AutoML (v4.0.1) [23] is another suite of AutoML models from Google Cloud which provides a user-friendly interface for training, deploying, and managing ML models. It leverages Google’s state-of-the-art neural architecture search (NAS) technology to automatically discover the best neural network architecture for a given task. Google AutoML supports various applications, including image classification, object detection, natural language processing, and more. It is designed to democratize ML by making it accessible to users with limited expertise in data science and machine learning. The Tree-based Pipeline Optimization Tool (TPOT) (v1.0.0) [24] is another AutoML tool that stands out due to its inherent approach to pipeline optimization. By leveraging genetic programming, TPOT can discover and optimize complex pipelines that would be difficult for human data scientists to design manually. This makes TPOT particularly useful for tasks that involve high-dimensional data and complex feature interactions.

While existing methodologies offer diverse approaches for feature extraction and model construction, they often lack seamless integration and intuitive interfaces, hindering widespread adoption and real-world applicability. As such, there remains a gap in the availability of a user-friendly, comprehensive tool that addresses methodological complexities, interpretability concerns, and bias identification through an all-in-one framework. Specifically, for the diagnosis of csPCa by MRI, existing methods face limitations in clinical adoption due to complex model tuning, lack of explainability, and limited generalizability across multi-vendor datasets. Within this context, the present study introduces Simplatab, a holistic solution with innovative methodologies to streamline the radiomics workflow in the context of PCa diagnosis, while prioritizing transparency, interpretability, and bias assessment to enhance the diagnostic process. Simplatab addresses these gaps by offering an end-to-end, no-code AutoML framework that integrates bias detection, automated feature selection, and explainability tools. Unlike traditional AI models that require expert curation, Simplatab facilitates unbiased, transparent, and scalable radiomics-based prostate cancer detection, enhancing its applicability in clinical practice. It is important to note that Simplatab is not limited to PCa diagnosis, but it preserves the same functionality for a variety of ML problems and features provided in tabular format.

The main contributions of this study are:

1. The provision of an open-source automated ML Framework with XAI analysis included, that does not require any code capabilities from the end-user. It encompasses functionalities for data bias detection, feature selection, ML algorithm selection, and hyperparameter optimization, either exhaustive or randomized. An easily interpretable, complete report with the trained models as pickle files and their performance is given as Excel files and images, including ROC-AUC curves, precision–recall curves, and results for both internal K-fold and external validation using six performance metrics (Figure S1). Furthermore, Shapley analysis results for feature importance are provided along with a bias assessment figures to facilitate the detection of bias and notify the user of it. Additionally, a model vulnerabilities detection model is included to inform the user regarding models’ deficiencies related to performance bias, robustness, calibration (overconfident and underconfident predictions), data leakage, stochasticity, and confounded features presence.
2. The evaluation of Simplatab in the context of radiomics-based analysis of clinically significant prostate cancer (csPCa) using bi-parametric MR images of 4816 patients from twelve clinical centers in nine European countries.
3. The assessment of radiomic features and the identification of the most valuable ones for predicting csPCA from bi-parametric MR images.
4. The assessment of Simplatab’s versality by experimenting on two additional use cases, presented in Appendix A (Bank Marketing Campaign Strategies) and in Appendix B (Airline Customer Satisfaction).

## 2. Materials and Methods

### 2.1. Simplatab Description

**Figure 1:**
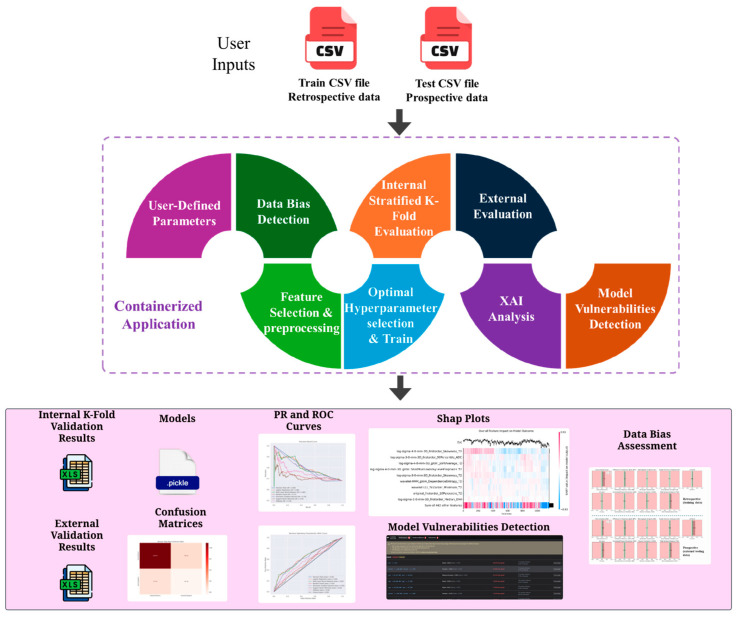
Schematic representation of Simplatab AutoML framework.

Figure 1 illustrates the complete representation and functionalities of the proposed tool as a containerized application. The user must define two files within the input folder, the Train CSV file and the Test CSV file. These files should contain columns for features and a target column from which the user intends the tool to train and predict. The Train CSV file will be partitioned in a stratified manner based on the number of K-Folds defined by the user. Subsequently, the tool will determine the hyperparameters if the user desires to perform hyperparameter optimization; otherwise, the training will proceed using the default parameters for each model, ensuring a faster response time.

Table 1 presents the algorithmic pipeline of Simplatab. For the use case of radiomics-based analysis, it begins by loading and preprocessing the training $\left(Xtrain,ytrain\right)$ and testing $\left(Xtest,ytest\right)$ datasets from CSV files and extracting hyperparameters and model settings P from an automatically generated YAML file. This file is configured from the end user’s selections from the HTML front-end. In the next step, if the user has selected to perform bias assessment (DB as logical value), then the bias assessment module will run, and it will produce the bias measurements for both the training and testing sets for a specified categorical feature Feat, in the form of JSON files and summary plots. Afterwards, for each model m∈M, the training data from each fold k are first preprocessed and undergo feature selection $F_{m}\left(X_{train}^{k},y_{train}^{k}\right)$, and this model m is then initialized with each configuration of hyperparameters $H_{i}$. The model is trained using K-fold cross-validation, where for each fold k, the model ${\hat{\;F}}_{m_{H_{i}}}^{k}$ is trained, optimized for threshold ${\;T}_{m_{H_{i}}}^{k}$ based on user-selected metric G, and evaluated, resulting in $E_{m_{H_{i}}}^{k}$. G is adjusted by the end user to suit specific clinical contexts. For instance, although a high sensitivity minimizes missed diagnoses, its lower specificity may increase false positives; thus, the framework permits recalibration of the threshold to achieve a balanced trade-off between sensitivity and specificity in line with clinical risk–benefit considerations. The average evaluation $E_{m_{H^{OPTIMAL}}}$ is computed across folds, mean and standard deviation results are saved to an Excel file, and confusion matrices are generated. After testing each hyperparameter configuration for a specific grid, the best model is selected. During external validation, each model m∈M is trained on the entire training set, utilizing the optimal hyperparameters $H^{optimal}$ found on the internal validation scheme, evaluated on the test set $E_{m}^{test}$, with the average threshold ${\;T}_{m_{H^{optimal}}}^{average}$ obtained from each fold and analyzed using Shapley values, $S_{m}$. ROC-AUC curves, precision–recall curves, and SHAP plots are generated. Furthermore, the trained model is assessed for potential vulnerabilities utilizing Simplatab’s “Model Vulnerability Detection” module that is applied in the testing dataset. Finally, results including evaluation metrics for both the internal K-fold and the external evaluation are produced as Excel files, SHAP plots are exported, and trained models $\hat{F}$ are saved as pickle files. A visual representation of the outputs is given in the Supplementary Materials, Figure S1.

### 2.2. Data Bias Detection Module

The DBD (data bias detection) toolkit provides a set of metrics for detecting biases in datasets, especially focusing on facets like gender and outcomes such as disease status. The toolkit employs a suite of statistical-based metrics to offer a holistic view of data bias before the AI model training process. The toolkit also supports cluster analysis using the MiniSOM clustering algorithm to identify and analyze biases within clusters. In this case, the optimal number of clusters is determined by selecting the cluster having the highest Davies–Bouldin (DB) score across a series of predefined clusters under evaluation. Then, the metrics are calculated, per identified cluster, to detect biases in subsets of the original data which might not be detected in the whole dataset. This supports the following metrics [25,26]: (i) the class imbalance (CI), which evaluates the imbalance between the groups within a facet; (ii) the difference in proportions of labels (DPL), which measures the disparity in positive outcomes between the groups in the facet; (iii) the demographic disparity (DD), which computes the disparity for specific groups in the facet; (iv) the Jensen–Shannon (JS) divergence, which is similar to Kullback–Leibler (KL) but a symmetrized version; (v) the total variation distance (TVD), which measures the distance between distributions of facets and outcomes; (vi) the Kolmogorov–Smirnov (KS) metric, which assesses the statistical distance between distributions; (vii) the normalized mutual information (NMI), which measures the information shared between categorical variables, normalized over possible outcomes; (viii) the Pearson correlation (CORR), which determines the linear correlation between the facet and the outcome; and (ix) the logistic regression (LR) coefficient, which assesses the influence of the facet on the outcome through a logistic regression model. The toolkit provides output plots for visualizing each metric along with the fairness and bias decision boundaries, as well as a JSON file with the values per metric. Any detected biases are reported to the user for further consideration.

### 2.3. Model Vulnerabilities Detection Module

The model vulnerability detection module in Simplatab, utilizing the Giskard library [27], is designed to ensure reliability and integrity of machine learning models. This module includes several model evaluation tests which are essential for safeguarding the model’s performance and trustworthiness, especially in clinical applications. These tests allow the detection of potential deficiencies in models by assessing (i) performance bias, (ii) robustness, (iii) calibration, (iv) data leakage, (v) stochasticity, and (vi) confounded features with respect to the target outcome. For instance, performance bias identifies disparities arising from imbalanced data, systemic biases, or underrepresented features. Robustness tests the model’s resilience against perturbations (for example, changing categorical encoding strategy) and noise, crucial for generalizing across a variety of cases. Calibration, which consists of both overconfidence and underconfidence measurements, detects whether there are features affecting the models’ outcomes towards the wrong prediction with high probability or whether the probability is close to random chance. Furthermore, data leakage detection identifies whether external information is present which leads to falsely better results than expected. Stochasticity analysis examines variability due to random processes in training, and whether the results remain unchanged with the same data assessed multiple times. If there is a significant deviation in the results, then the model has not learned the desired feature patterns. Lastly, identifying and mitigating spurious correlations ensures that the model learns meaningful patterns, rather than coincidental associations. For instance, there may be confounding features that, while closely related to the outcome, must be discarded from the analysis as they do not represent realistic scenarios or were never intended to be part of the problem formulation.

### 2.4. Dataset Description and Preprocessing

In our study, we utilized data provided by 4816 patients from the ProstateNet database. The ProstateNet dataset consists of bi-parametric MRIs (T2w, ADC, DWI) from 13 clinical centers and 4 vendors (Siemens, Philips, GE, Toshiba). Specifically, the data across clinical centers were distributed as follows: 1252 cases from RadboudUMC (Nijmegen, The Netherlands), 662 cases from Champalimaud (Lisboa, Portugal), 575 cases from RMH (UK), 626 cases from NCI (Vilnius, Lithuania), 517 cases from Haceteppe (Ankara, Turkey), 264 cases from IPC (Marseille, France), 252 cases from IDIBGI (Girona, Spain), 296 cases from HULAFE (Valencia, Spain), 148 cases from QUIRONSALUD (Across Spain), 78 cases from FPO (Turin, Italy), 52 cases from JCC (Across Portugal), 12 cases from UNIPI (Pisa, Italy), and 83 cases from GAONA (Athens, Greece). Furthermore, the clinical eligibility criteria for the analysis included histological confirmation from either biopsy or prostatectomy. Moreover, the number of cases per MR vendor was: (i) 1119 cases from General Electric, (ii) 1749 cases from Philips, (iii) 1940 cases from Siemens, and (iv) 9 cases from Toshiba.

For the csPCA detection from the prostate’s peripheral zone (PZ), a series of preprocessing operations was performed before extracting the radiomics from T2-weighted, ADC, and DWI MR sequences. Furthermore, the model was trained in 3 MR vendors, namely, Siemens, Philips, and General Electric. The segmentation results were post-processed with opening and closing operations to ensure the reliability of the PZ mask outcomes.

Additionally, for the radiomics extraction process, different configurations were selected for each MR sequence separately. Since we dealt with a multi-vendor and multi-centric dataset, it was important to perform preprocessing stages that would provide better uniformity for radiomics features. To deal with the non-uniformities introduced by low-frequency intensities present in MRIs, the N4 Bias Field Correction was applied to all images [28,29]. Afterwards, the PyRadiomics library (version 3.1.0a2) was used for feature extraction, with the configuration settings set to default, with the sole exception of the fixed bin-width discretization, which was adapted for every MR image separately. The selected width should generate a histogram for each image with a range from 30 to 128 bins. In the end, we obtained a dataset with 3 types of MRI sequences (T2, ADC, DWI) from the prostate’s PZ.

Furthermore, the feature selection process comprises several sequential steps. Initially, features exhibiting high inter-correlation are excluded based on their correlation coefficients, with the threshold—ranging from −1 to 1—being user-defined through Simplatab’s interface. Subsequently, a consensus approach is employed, combining the SULOV method with recursive feature elimination (RFE) [30], which utilizes the XGBoost model as the underlying evaluation model, to determine the most significant features.

### 2.5. Machine Learning Algorithms

Simplatab includes seven classifiers—Logistic Regression, Decision Tree, Random Forest, XGBoost, Multi-layer Perceptron, Stochastic Gradient Descent, and Support Vector Machines, selected due to their proven effectiveness on tabular radiomics data, offering a balance of interpretability, efficiency, and predictive performance [31,32]. The Decision Tree (DT) classifier is a computationally efficient non-parametric algorithm that models decisions as a tree-like structure of nodes, where each internal node represents a feature, each branch represents a decision rule, and each leaf node represents an outcome, typically using metrics like Gini impurity or entropy. The Logistic Regression (LR) classifier estimates the probability that a given input belongs to a particular class by applying the logistic sigmoid function to a linear combination of input features to optimize the log-likelihood function, using techniques such as gradient descent. The Multi-layer Perceptron (MLP) classifier uses non-linear activation functions (e.g., ReLU, sigmoid, tanh) to learn complex patterns in the data, and they are trained using backpropagation to minimize error by adjusting the weights through gradient descent. The Random Forest (RF) algorithm is an ensemble learning method that constructs a multitude of decision trees during training and outputs the mode of the classes of the individual trees by considering a random subset of features for each split. Simplatab also supports the Stochastic Gradient Descent (SGD) optimization algorithm, which updates the model parameters incrementally by computing the gradient of the loss function for each training example (or a small batch). It also supports the Support Vector Machines (SVM) algorithm, which constructs a hyperplane or set of hyperplanes in a high-dimensional space that maximally separates csPCa from non-csPCa. The SVM can also be extended to handle non-linear boundaries using kernel functions like the polynomial, radial basis function (RBF), and sigmoid kernels. Finally, Simplatab supports the XGBoost (Extreme Gradient Boosting) algorithm, which is an advanced implementation of gradient boosting designed for performance and speed by building an ensemble of weak prediction models, typically decision trees, in a sequential manner, where each subsequent tree is trained to correct the errors of its predecessors.

### 2.6. Evaluation Scheme

In our analyses, we partitioned the overall dataset in the retrospective training set consisting of 3656 patients (Train.csv) and the prospective external validation set consisting of 1162 patients (Test.csv). For optimal hyperparameter selection and internal validation purposes, a 10-fold stratified cross-validation scheme was performed, partitioning the training set to 10 sub-train and validation sets, each one consisting of 3291 and 365 cases, respectively, for each fold. On each fold, feature selection and data scaling were performed on the sub-train sets and later were applied on the sub-validation sets. For external validation, the selected hyperparameters and the thresholds extracted from the internal 10-fold cross-validation were utilized to retrain the models on the whole retrospective set, while the results were obtained on the prospective set. Furthermoer, we utilized 6 metrics to assess the performance of the models, (i) sensitivity, (ii) specificity, (iii) AUC score, (iv) F-score, (v) accuracy, and (vi) balanced accuracy.

### 2.7. Open Access Repository, Container Application, and Community Support

**Figure 2:**
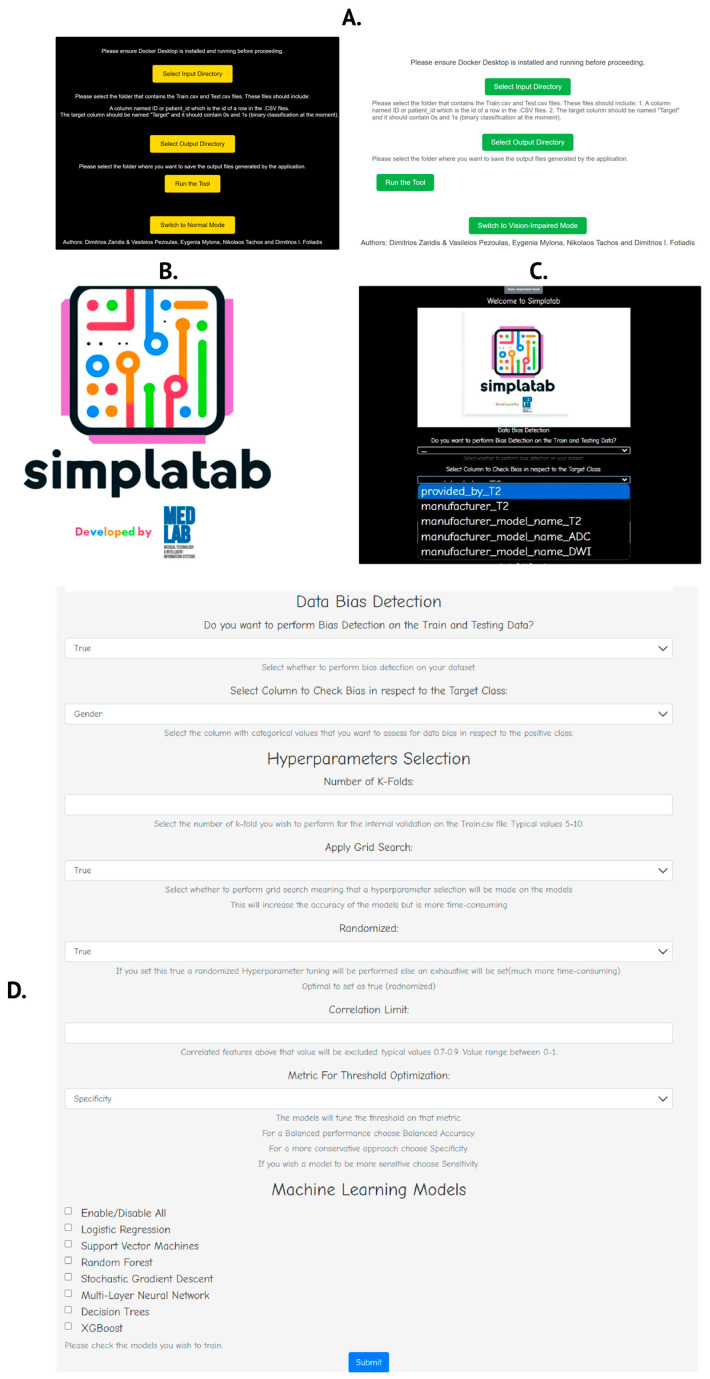
(**A**) Desktop app, (**B**) introduction page, (**C**) introduction page for individuals with vision impairment, and (**D**) the parameter selection from the front-end.

Simplatab offers two modes of operation. The most convenient method is as a containerized application available on Docker Hub under the name dimzaridis/simplatab-machine-learning-automator. Comprehensive documentation is provided there, including instructions on how to download and run it with a single command. Additionally, a GitHub (v1.0.0) repository is available for contributions or direct use, with the necessary documentation for local installation. In particular, in the GitHub repository, the Simplatab.EXE file serves as a Desktop App for easy execution. Currently, when the user runs the tool, it redirects to localhost on port 5000 (which can be reconfigured) and allows parameter selection via a front-end interface created with HTML. The only requirement for the user is to install Docker Desktop locally, which includes a GUI, and to provide two input files when prompted by the desktop app, Train.csv (retrospective set) and Test.csv (prospective set), to run the experiment. For the GitHub version, the user can directly clone the repository and build the docker if they so desire, or they may run the python API which is also offered. Furthermore, we have added several continuous integration (CI) automations for unit testing and continuous deployment (CD) automations for building the docker image from the main branch automatically. In this manner, the contribution of the community may be more practical for continuous integration and deployment. In Figure 2B, the developed HTML-based front-end is presented. We tried to keep it as simple as possible, especially for non-experienced users, including only a drop-down menu and logical values (True/False). Therefore, the front-end mechanism facilitates the easy yet effective execution of the framework. In Figure 2A, the desktop app interface is presented, where the users select their input and output folders. After selecting to run the tool, users are redirected to a locally executed HTML front-end to proceed with parameter selections. Furthermore, we strive to promote inclusiveness by adding a functionality option for vision-impaired individuals as shown in Figure 2C, while for each fillable section, we explain the function of the section, typical values for free text sections, and the advantages and disadvantages of using a specific mechanism. For instance, if the user opts to perform grid search, an indication is written below to notify them that this is a much more time-consuming process.

## 3. Results

### 3.1. Bias Detection Assessment

**Figure 3:**
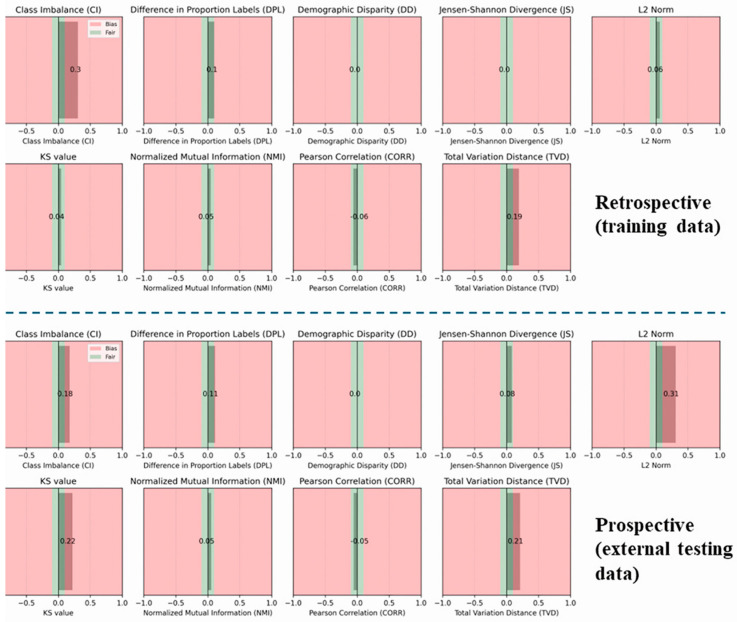
Bias assessment using nine metrics with respect to different MR vendors (Siemens, Phillips, General Electric, and Toshiba) and target class (csPCa) for the retrospective and the prospective sets.

In Figure 3, we present the bias assessment for different MR vendors regarding the non-csPCa and csPCa classes, using both retrospective and prospective datasets. Green areas denote fair data, red areas indicate bias, and gray zones represent the computed metrics under the current scenario. A critical metric, DPL, measures class imbalance across different vendors, as illustrated in the figure. The distribution of MR vendors for the two classes is either fair or marginally biased for both datasets. The CI metric suggests bias, indicating that several MR vendors are underrepresented (General Electric: 1119 cases, Philips: 1749 cases, Siemens: 1940 cases, Toshiba: 9 cases). Conversely, the KS (Kolmogorov–Smirnov) statistical test shows that the maximum differences between the cumulative distributions of the vendors deviate from the fair area for prospective data but remain within the fair area for retrospective data. Additionally, the TVD indicates that the probability distributions of each vendor concerning the target classes diverge from each other, suggesting a minor bias for some vendors. For example, the Toshiba vendor, with only nine cases, exhibits a higher likelihood of imbalanced samples between classes, impacting the metrics significantly. Entropy-based metrics, JS and NMI, demonstrate that both sets are fair in terms of entropy-related measurements. Overall, both the retrospective and prospective datasets proved to be fair with respect to the MRI vendor.

### 3.2. Internal Stratified 10-Fold Cross-Validation

The internal stratified 10-fold cross-validation results for various machine learning models are summarized in Table 2. SVM demonstrated the highest sensitivity (0.78 ± 0.04) but had lower specificity (0.56 ± 0.06). XGBoost showed strong overall performance, with high AUC (0.77 ± 0.03) and balanced accuracy (0.71 ± 0.02). LR, RF, and SGD displayed comparable results, with balanced accuracies around 0.69–0.70 and AUC values ranging from 0.73 to 0.77. MLP performed comparably, with balanced accuracy (0.68 ± 0.03) and AUC (0.72 ± 0.03). DT, although less effective overall, showed balanced accuracy (0.65 ± 0.03). Overall, RF and XGBoost provided the most robust and balanced performance across the evaluated metrics. Moreover, the 10-fold cross-validation performance for the same models trained solely on clinical variables available in the dataset are presented in Appendix C, Table A5.

### 3.3. External Validation

The external validation results for various machine learning models are summarized in Table 3. SVM demonstrated the highest sensitivity (0.87) but had lower specificity (0.44). XGBoost showed a strong overall performance, with high AUC (0.74) and balanced accuracy (0.67). RF provided a well-balanced performance, with a balanced accuracy of 0.68 and an AUC of 0.73. LR and SGD displayed comparable results, with balanced accuracies around 0.65 and AUC values ranging from 0.71 to 0.72. MLP performed comparably, with a balanced accuracy of 0.64 and an AUC of 0.71. DT, although less effective overall, showed a balanced accuracy of 0.62. Overall, RF and XGBoost provided the most robust and balanced performance across the evaluated metrics. Furthermore, a statistical significance comparison between the top two best-performing models—XGBoost and Random Forest—was conducted using the Wilcoxon signed-rank test. The test yielded a *p*-value of 0.06, which is marginally above the conventional significance threshold (α = 0.05), suggesting that the performance difference between the two models is not statistically significant. Additionally, the evaluation using the prospective dataset for the same models trained solely on clinical variables available in the dataset is presented in Appendix C, Table A6.

**Figure 4:**
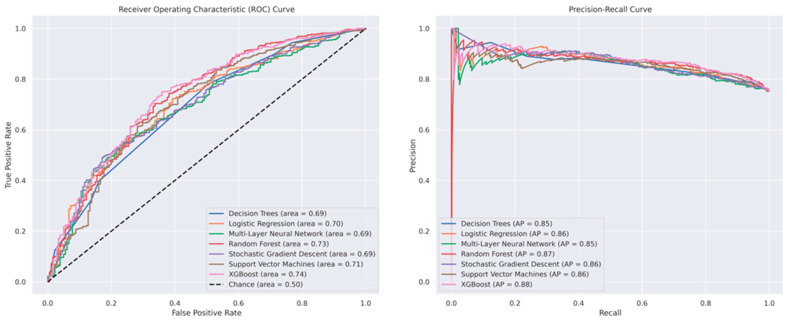
AUC-ROC (**left**) and precision–recall curves (**right**) for the prospective dataset.

Figure 4 illustrates the AUC-ROC and PR curves. In the ROC curve plot, the XGBoost model demonstrated the highest area under the curve (AUC) of 0.74, indicating strong overall performance. This is followed closely by RF and SVM, with AUCs of 0.73 and 0.71, respectively. LR and SGD both achieved an AUC of 0.70, while DT and MLP each had an AUC of 0.69. The PR curve plot shows that XGBoost again performed well, achieving the highest average precision (AP) of 0.88, which is slightly better than RF with an AP of 0.87. LR, SVM, and SGD each had an AP of 0.86, demonstrating competitive performance. DT and MLP both had an AP of 0.85. Additionally, in Appendix C, Table A7, we have conducted a post hoc analysis of MR vendor variations with the top two best-performing models.

### 3.4. XAI Analysis

**Figure 5:**
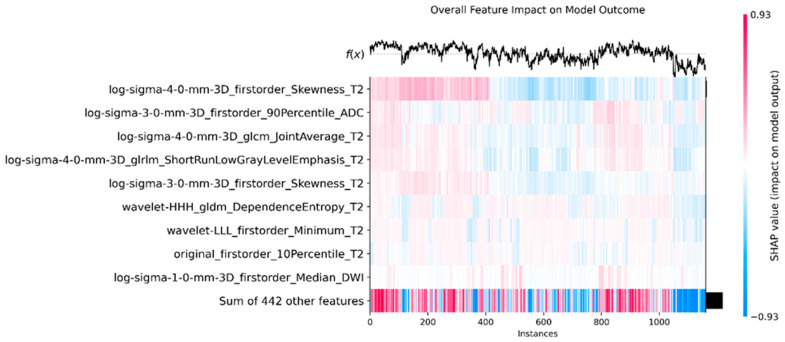
Heatmap plot with the SHAP values for each feature ordered by importance, correlated with the XGBoost outcome, for the external dataset.

The SHAP heatmap plot, presented in Figure 5, shows how several radiomic features affect XGBoost. Specifically, it quantifies the contribution of individual radiomic features to the model’s predictions across different cases. Rows denote the different radiomic features, while columns represent each individual patient used in the dataset. The color intensity in the heatmap represents the SHAP value, which measures the importance of the feature for that instance with respect to the model’s outcome. Furthermore, positive influence is presented in red, while negative influence is represented in blue. Prominent features like “log-sigma-4-0-mm-3D_firstorder_Skewness_T2” and “log-sigma-3-0-mm-3D_firstorder_90Percentile_ADC” show a greater influence, as marked by color gradient. For instance, spikes in the SHAP values (as seen in red) indicate that greater skewness results in shifting the probabilities towards the csPCa class, while lesser skewness (in blue) shifts the model’s outcome towards the control class. Moreover, the last row (sum of 442 other features) has an aggregated SHAP value for all the remaining features that individually may not be significant but altogether play a crucial role in shifting the model’s outcomes. This implies that there is no clear radiomic feature that discriminates the problem itself, but rather a combination of all the features may contribute significantly to csPCa detection.

### 3.5. Interpretation of Important Features

**Figure 6:**
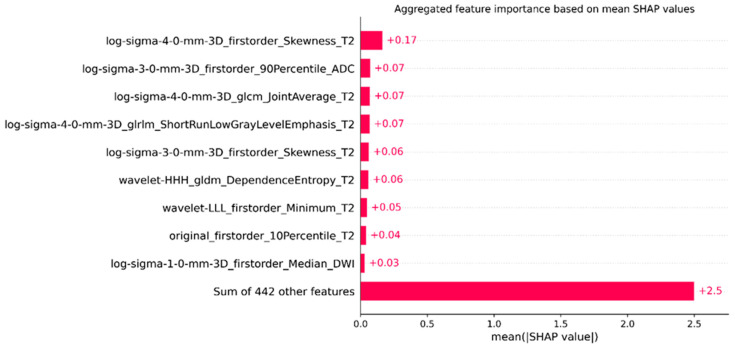
Feature importance in the XGBoost model, for the external dataset.

Figure 6 presents the aggregated feature importance of each radiomic feature in XGBoost. A brief description follows to map those features to their physical meanings. First-order statistics refer to fundamental statistical measures of the intensity values in the volume (mean voxel intensity, median, etc.). For example, “log-sigma-4-0-mm-3D_firstorder_Skewness_T2” represents the skewness of voxel intensities after applying a Laplacian filter in the T2 sequence. Similarly, “log-sigma-3-0-mm-3D_firstorder_90Percentile_ADC” refers to the 90th percentile of voxel intensities, reflecting higher intensities in the ADC sequence. GLCM features, such as “log-sigma-4-0-mm-3D_glcm_JointAverage_T2”, quantify texture information by evaluating the spatial correlation between pairs of voxels. This feature captures the average of the joint probability distribution of pairs of voxels, measuring texture uniformity. The GLRLM feature, “log-sigma-4-0-mm-3D_glrlm_ShortRunLowGrayLevelEmphasis_T2”, quantifies the structure of brief sequences of adjacent voxels with low intensity in the T2 sequence, indicating texture patterns characterized by short runs of similar gray levels. Additional texture information is obtained from features like “wavelet-HHH_gldm_DependenceEntropy_T2”, which measures the entropy of dependence within the gray level dependence matrix in the T2 sequence, capturing the complexity and heterogeneity of textures. “Wavelet-LLL_firstorder_Minimum_T2” represents the minimum intensity value after applying a wavelet filter, indicating the darkest regions within the volume. The SHAP analysis highlights the significance of both basic statistical characteristics and more complex textural characteristics derived from various MR sequences. For instance, T2- and ADC-derived metrics play a crucial role in differentiating csPCa patients. “Original_firstorder_10Percentile_T2” measures the 10th percentile of voxel intensities, capturing the lower values of the intensity distribution, while “log-sigma-1-0-mm-3D_firstorder_Median_DWI” reflects the median intensity value, indicating central tendency within patients. This analysis highlights the significance of both basic statistical characteristics and more complex textural characteristics derived from different MR sequences, with a focus on T2- and ADC-derived metrics, while all the other 442 features also contribute significantly in differentiating csPCa patients.

### 3.6. Model Vulnerability Detection Results

Table 4 presents the results of the model vulnerability analysis (XGBoost), revealing that specific radiomic features significantly impact overconfidence rates and performance metrics in predictions. Notably, features such as “log-sigma-4-0-mm-3D_firstorder_Skewness_T2” and “wavelet-LLL_firstorder_Minimum_T2” showed an overconfidence rate increase of 40.38% and 32.80%, respectively, compared to global rates. This indicates a high frequency of overconfident incorrect predictions when these features fall below certain thresholds. Similarly, “original_firstorder_10Percentile_T2” and “log-sigma-3-0-mm-3D_firstorder_Skewness_T2” were associated with significant overconfidence, suggesting that these features exacerbate model vulnerability in specific data slices.

More specifically, for some records in the dataset where “log-sigma-4-0-mm-3D_firstorder_Skewness_T2” is less than 0.9229, there was a significantly higher number of overconfident wrong predictions, with 81 samples corresponding to 81.82% of the wrong predictions in this data slice. Similarly, for “wavelet-LLL_firstorder_Minimum_T2” values less than 71.82, there were 137 samples, accounting for 77.40% of the wrong predictions. For “original_firstorder_10Percentile_T2” values less than 29.361, there were 116 samples, corresponding to 77.33% of the wrong predictions. Finally, for “log-sigma-3-0-mm-3D_firstorder_Skewness_T2” values less than 0.9336, there were 89 samples, making up 76.07% of the wrong predictions.

On the other hand, features such as “log-sigma-4-0-mm-3D_glszm_SmallAreaEmphasis_T2” were linked to decreased balanced accuracy. For feature values greater than or equal to 0.417, the balanced accuracy was 13.14% lower than the global balanced accuracy. Furthermore, “feature wavelet-LLL_glcm_Imc1_DWI” showed a reduced precision, with values below 0.1527 leading to a precision rate 7.71% lower than the global precision rate, and balanced accuracy 6.45% lower for values less than or equal to 0.1615.

## 4. Discussion

In this work, we propose Simplatab, an AutoML framework designed to enable end-users to automatically develop a straightforward and robust ML pipeline without prior coding knowledge, using a user-friendly interface for parameter selection. The framework outputs are exported in a human-understandable format, translating into Excel files and plots in an internal and external validation scheme. Additionally, Simplatab offers XAI analysis to identify important features, ensuring that they align with expected features, while a bias detection module is also included to assess whether bias exists with respect to specific characteristics of the given dataset (csPCa detection in our case). Moreover, Simplatab offers a report regarding potential vulnerabilities of the tested models, such as (i) performance bias, (ii) data leakage, (iii) robustness, (iv) stochasticity, (v) calibration issues, and (vi) confounded features.

Furthermore, we conducted a csPCa radiomics-based analysis on a pan-European cohort of 4816 patients across 12 clinical centers using bi-parametric MR images. We also assessed the impact of radiomic features on the predictive models tested for this cohort, allowing us to identify the most valuable features for predicting csPCa. Although no single radiomic feature is fully predictive, a combination of specific features significantly enabled the differentiation between csPCa and non-csPCa cases. Clinically, high skewness values in ADC-derived features (‘log-sigma-3-0-mm-3D_firstorder_90Percentile_ADC’) suggest areas of restricted diffusion, a hallmark of malignancy. Similarly, texture-based features such as ‘log-sigma-4-0-mm-3D_firstorder_Skewness_T2’ highlight intra-lesional heterogeneity, which may indicate aggressive tumor behavior. These insights can be integrated into radiologists’ decision-making processes, particularly for lesions that appear indeterminate in conventional MRI assessment, thereby refining risk stratification and biopsy recommendations.

On the whole, Simplatab is a general-purpose AutoML tool that could be utilized for a variety of ML tasks; two additional use cases have been executed and presented in Appendix A and Appendix B, in addition to the csPCa radiomics-based detection, to support that claim.

Table 5 presents a comparison between the features of the proposed Simplatab AutoML tool and other similar tools. The primary advantage of Simplatab lies in its inclusion of Shapley XAI analysis and feature importance, data bias detection, model vulnerabilities detection, and ease of use for non-expert users. It enables users to run models and obtain various reporting items, such as: (i) ROC-AUC and PR curves, (ii) two Excel files with scores from internal K-fold and external validation, (iii) confusion matrices for both internal and external validation, (iv) SHAP plots for each tested algorithm on the external set, (v) bias indicators for the train and test sets defined by the user, (vi) bundled pipelines as pickle files for separate use, and (vii) vulnerabilities of the model and corresponding features. This tool enhances AutoML applications, particularly in biomedical data analysis, where Shapley analysis [33] and thorough hyperparameter optimization [34] are crucial for identifying important features. Regarding the other AutoML frameworks in comparison, the Auto-WEKA integrates the WEKA platform and automates model selection and hyperparameter optimization using Bayesian optimization. It provides open access and community support, making it suitable for novice users. Nevertheless, despite its robust optimization capabilities, Auto-WEKA lacks advanced user interface features and is less user-friendly compared to other tools. On the other hand, Auto-Sklearn builds upon the scikit-learn library, offering automated model selection, hyperparameter optimization, and support for external validation. It provides advanced customization and extensive community support. However, the tool requires moderate coding knowledge, which can be a barrier for non-technical users, and its setup and configuration can be complex. Furthermore, ML-Plan provides a comprehensive range of selectable machine learning models and advanced customization options. It supports detailed reporting and external validation, making it suitable for sophisticated machine learning tasks. However, the setup process is also complex, posing challenges for users without a technical background, and it requires more exhaustive manual configuration compared to other tools. Moreover, ATM offers cloud integration, facilitating the processing of large datasets. It supports detailed reporting, model export, and automatic hyperparameter tuning. Nevertheless, ATM lacks the ease of use and advanced visualization features found in some of its competitors, making it less accessible to non-expert users. Likewise, TPOT stands out for its ease of use and its hyperparameter identification via a tree-based optimization inspired by genetic programming. While TPOT presents a plethora of capabilities for AutoML development processes, it falls short in areas such as explainability analysis, data bias detection, and model vulnerabilities detection. Although it provides the best-performing model, it often overlooks the importance of incorporating user input in the selection of AI models. Conversely, Google AutoML provides a highly user-friendly, cloud-based platform with advanced visualization and comprehensive reporting capabilities. It simplifies the machine learning process for users with minimal technical expertise. However, the tool requires a subscription and does not allow open access, which limits its accessibility. Additionally, it poses potential security concerns for sensitive data due to cloud-based experimentations which pose the threat of leaking sensitive information from patients and institutions. Unlike conventional AI-based radiomics tools such as IBM Watson Health [35], QTIM [36], and Radiomics.io [37], which focus primarily on feature extraction and AI-based classification, Simplatab provides a fully automated pipeline that includes data bias detection, model vulnerability assessment, and integrated explainability via SHAP analysis. Most existing clinical AI-radiomics tools require manual feature engineering and extensive preprocessing, and lack robust interpretability mechanisms, making them challenging to integrate into real-world workflows. Simplatab addresses these gaps by automating the entire machine learning pipeline while ensuring fairness and explainability. Furthermore, the tool has been evaluated on a large, multi-vendor, multi-center prostate cancer dataset, making it more robust for clinical application than many single-center AI models.

It is of paramount importance to note that all the existing AutoML tools do not prioritize model explainability, interpretability, and data bias reporting, thus lacking XAI and data bias detection mechanisms such as the Shapley analyses and the DBD toolkit, which are encompassed by the Simplatab framework. For the latter, data bias detection is a crucial step to identify socio-economic, statistical, and other biases and address them properly to produce algorithms with increased inclusivity. In most biomedical applications, especially when addressing problems with clinical related data, feature importance and bias detection constitute the cornerstone of a healthcare-related stakeholder’s trust in a model’s usability and willingness to introduce it into clinical practice. In general, the main Simplatab features that extend the functionalities of current state-of-the-art AutoML tools are (i) usability with no prior code knowledge by the end user, (ii) data bias assessment, (iii) XAI analysis of a model’s outcomes, (iv) model vulnerabilities reporting, (v) a user-friendly interface, and (vi) human-understandable reporting.

The emergence of AI presents both tremendous opportunities and significant challenges. Building trustworthy AI requires a multifaceted approach, targeting distinct characteristics/dimensions of trustworthiness that are opposed by various factors [38]. Thus, an AI-based system should be (i) safe, (ii) secure and resilient, (iii) explainable and interpretable, (iv) privacy-enhanced, (v) valid and reliable, (vi) accountable and transparent, and (vii) fair (managing harmful bias) in order to produce trust. With that in mind, Simplatab provides functionalities to AI developers to ensure compliance with the various dimensions of trustworthiness. For instance, conducting XAI analysis and model vulnerability detection as a bundled module within the proposed framework for each tested model leverages the explainability and interpretability of an AI model. On the other hand, validity, reliability, and fairness are acquired through the integrated validation scheme (stratified K-fold), the external validation pipeline, and the calculation of various well-known evaluation metrics, while the bias assessment module provides a variety of metrics to assess bias presence in the dataset. In comparison with other relevant toolkits, Simplatab offers an automated ML development framework incorporating modules to build more trustworthy AI pipelines, as the majority of these toolkits solely automate the development of ML operations. This makes Simplatab particularly suitable for biomedical applications, where interpretability and bias detection are of paramount importance.

While our proposed AutoML framework is straightforward, several enhancements are planned for future development. For instance, we intend to incorporate experiment tracking libraries such as MLFlow [39] and Weights and Biases to store models and improve transparency. Additionally, a post-training model bias mitigation module, like those proposed by IBM’s AI Fairness 360 module [40], will be added. Furthermore, the tool will offer more configuration options for users, including additional models and support for meta-learning via stacking. Additionally, although Simplatab has been extensively tested on a large, multi-center MRI dataset, further real-world clinical validation is essential to assess its practical deployment in clinical workflows.

## 5. Conclusions

This study introduces Simplatab, an open-source AutoML framework designed to enable users to run an ML pipeline, perform XAI analysis, and measure data bias in a multifaceted manner, all without requiring prior coding knowledge. Simplatab prioritizes ease of use but also provides informative yet simple outcomes to effectively enhance the interpretability of models’ underlying mechanisms. For AI non-experts, Simplatab provides a range of insightful and comprehensible figures, including ROC-AUC curves, confusion matrices, detailed reports in Excel format for both internal and external validation, Shapley analysis, data bias assessment, and model vulnerabilities detection. The framework also provides trained bundled models for external use by the user. We evaluated Simplatab with a pan-European cohort of 4816 patients, utilizing data from ProstateNet. This dataset includes diverse information from multiple vendors and clinical centers across Europe, specifically for radiomics-based csPCa from the prostate’s peripheral zone features. This extensive evaluation of Simplatab demonstrated the effectiveness of the tool in handling complex tabular data and providing a thorough analysis with the press of a button.

## Figures and Tables

**Table 1:** Simplatab’s implemented algorithmic steps.

**A. Load Data** $\left(Xtrain,ytrain\right)\leftarrow read\left(Train.csv\right)$ (Xtest,ytest)←read(Test.csv) **B. Read User’s Configuration from the Provided Front-end** $P\leftarrow read\left(P_{yaml}\right)$ DB,Feat, H, C, M, G, K ←P **C. Bias Assessment** If DB ←True $\mathrm{Bias}\;\mathrm{metrics},\;\mathrm{bias}\;\mathrm{plots}\;\leftarrow \left(Xtrain^{Feat},\;ytrain^{Feat}\right),\;(Xtest^{Feat},ytest^{Feat})$ Save bias metrics as JSON files for each set Save bias plots as PNG images for each set **D. Train Models With K-Fold Cross-validation** For each model m∈M $For\;each\;set\;of\;hyperparameters\;H_{i}\;\in H\;$ For each fold k ∈K (i) Standard scaling and encoding of training data $\;\;\;\;\;\;\;\;\;\;\;\;(ii)\;Feature\;selection\;F_{m}(X_{train}^{k},\;y_{train}^{k})$ $(iii)\;Initialize\;model\;m_{H_{i}}$ $\left(iv)\;Train\;model{\hat{\;F}}_{m_{H_{i}}}^{k}\leftarrow m_{H_{i}}(X_{train}^{k},\;y_{train}^{k}\right)$ $\left(v\right)\;Optimize\;threshold{\;T}_{m_{H_{i}}}^{k}\leftarrow \;optimize\;({\hat{\;F}}_{m_{H_{i}}}^{k},\;X_{train}^{k},\;y_{train}^{k},\;G)$ ${\;\;\;\;\;\;\;\;\;\;\;\;(vi)\;Evaluate\;model\;E}_{m_{H_{i}}}^{k}\leftarrow \;\;Evaluate\;({\hat{F}}_{m_{H_{i}}}^{k},\;X_{train}^{k},\;y_{train}^{k},\;{\;T}_{m_{H_{i}}}^{k})$ Calculate mean and standard deviation across K−folds for the optimal hyperparameters $E_{m_{H^{OPTIMAL}}}\leftarrow \;\frac{1}{K}\sum_{k=1}^{K}E_{m_{H^{OPTIMAL}}}^{k}$ Save results to Excel and generate confusion matrices **E. External Validation** For each model m∈M (i)Train model on the whole with optimal Hyperparameters ${\hat{\;F}}_{m_{H^{optimal}}}\leftarrow m(X_{train},y_{train},H^{optimal})$ ${\;\;\;\;\;\;\;(ii)\;Evaluate\;model\;E}_{m}^{test}\leftarrow \;\;Evaluate\;({\hat{\;F}}_{m_{H^{optimal}}},\;X_{test},y_{test},\;{\;T}_{m_{H^{optimal}}}^{average})$ ${\;\;\;\;\;(iii)\;Compute\;SHAP\;values\;S}_{m}\leftarrow \;\;SHAP\;({\hat{\;F}}_{m_{H^{optimal}}},\;X_{test}\;)$ (iv) Generate AUC−ROC and Precision−Recall curves, SHAP plots including radar, bar, heatmap plots $\left(v)\;\mathrm{Model}\;\mathrm{Vulnerability}\;\mathrm{Detection}\;\leftarrow \;\mathrm{DetVuln}(,\;X_{test},y_{test},{\hat{\;F}}_{m_{H^{optimal}}}\right)$ $\left(vi\right)Save\;results\;as\;Excel\;files,\;curves\;and\;plots\;as\;png\;images,$ and trained models as pickle files

**Table 2:** Ten-fold cross-validation results for the retrospective dataset.

Model	Sensitivity	Specificity	AUC	F-Score	Accuracy	Balanced Accuracy
Decision Trees	0.58 ± 0.10	0.72 ± 0.12	0.69 ± 0.03	0.69 ± 0.07	0.61 ± 0.05	0.65 ± 0.03
Logistic Regression	0.68 ± 0.08	0.70 ± 0.10	0.76 ± 0.02	0.77 ± 0.04	0.69 ± 0.04	0.69 ± 0.03
Multi-Layer Neural Network	0.69 ± 0.09	0.67 ± 0.10	0.72 ± 0.03	0.76 ± 0.05	0.68 ± 0.05	0.68 ± 0.03
Random Forest	0.70 ± 0.08	0.70 ± 0.07	0.77 ± 0.03	0.77 ± 0.05	0.70 ± 0.05	0.70 ± 0.02
Stochastic Gradient Descent	0.67 ± 0.07	0.70 ± 0.09	0.73 ± 0.02	0.76 ± 0.04	0.68 ± 0.04	0.69 ± 0.02
Support Vector Machines	0.78 ± 0.04	0.56 ± 0.06	0.73 ± 0.03	0.81 ± 0.02	0.73 ± 0.03	0.67 ± 0.03
XGBoost	0.74 ± 0.06	0.68 ± 0.06	0.77 ± 0.03	0.80 ± 0.04	0.72 ± 0.04	0.71 ± 0.02

**Table 3:** External validation results for the prospective dataset.

Model	Sensitivity	Specificity	AUC	F-Score	Accuracy	Balanced Accuracy
Decision Trees	0.67	0.56	0.66	0.72	0.64	0.62
Logistic Regression	0.76	0.54	0.72	0.77	0.69	0.65
Multi-Layer Neural Network	0.82	0.46	0.71	0.79	0.70	0.64
Random Forest	0.75	0.61	0.73	0.78	0.71	0.68
Stochastic Gradient Descent	0.68	0.61	0.71	0.73	0.66	0.65
Support Vector Machines	0.87	0.44	0.71	0.82	0.74	0.66
XGBoost	0.77	0.56	0.74	0.78	0.70	0.67

**Table 4:** XGBoost feature vulnerability report.

Feature	Metric	Deviation	Description
**‘log-sigma-4-0-mm-3D_firstorder_Skewness_T2’** **≤** **9.229 × 10^−1^**	Overconfidence rate	+40.38% than global	For records in the dataset where ‘log-sigma-4-0-mm-3D_firstorder_Skewness_T2’ < −9.229 × 10^−1^, we found a significantly higher number of overconfident wrong predictions (81 samples, corresponding to 81.82% of the wrong predictions in the data slice).
**‘wavelet-LLL_firstorder_Minimum_T2’** **≤** **7.182 × 10^1^**	Overconfidence rate	+32.80% than global	For records in the dataset where ‘wavelet-LLL_firstorder_Minimum_T2’ < −7.182 × 10^1^, we found a significantly higher number of overconfident wrong predictions (137 samples, corresponding to 77.40% of the wrong predictions in the data slice).
**‘original_firstorder_10Percentile_T2’ < 29.361**	Overconfidence rate	+32.69% than global	For records in the dataset where ‘original_firstorder_10Percentile_T2’ < 29.361, we found a significantly higher number of overconfident wrong predictions (116 samples, corresponding to 77.33% of the wrong predictions in the data slice).
**‘log-sigma-3-0-mm-3D_firstorder_Skewness_T2’** **≤** **9.336 × 10^−1^**	Overconfidence rate	+30.52% than global	For records in the dataset where ‘log-sigma-3-0-mm-3D_firstorder_Skewness_T2’ < −9.336 × 10^−1^, we found a significantly higher number of overconfident wrong predictions (89 samples, corresponding to 76.07% of the wrong predictions in the data slice).
**‘wavelet-HLL_glcm_Correlation_DWI’** **≤** **0.269**	Overconfidence rate	+22.03% than global	For records in the dataset where ‘wavelet-HLL_glcm_Correlation_DWI’ < 0.269, we found a significantly higher number of overconfident wrong predictions (133 samples, corresponding to 71.12% of the wrong predictions in the data slice).
**‘log-sigma-4-0-mm-3D_glszm_SmallAreaEmphasis_T2’ ≥ 0.222**	Overconfidence rate	+19.15% than global	For records in the dataset where ‘log-sigma-4-0-mm-3D_glszm_SmallAreaEmphasis_T2’ ≥ 0.222, we found a significantly higher number of overconfident wrong predictions (125 samples, corresponding to 69.44% of the wrong predictions in the data slice).
**‘wavelet-LLL_glcm_Imc1_DWI’** **≤** **1.527 × 10^−1^**	Overconfidence rate	+18.69% than global	For records in the dataset where ‘wavelet-LLL_glcm_Imc1_DWI’ < −1.527 × 10^−1^, we found a significantly higher number of overconfident wrong predictions (92 samples, corresponding to 69.17% of the wrong predictions in the data slice).
**‘log-sigma-3-0-mm-3D_firstorder_90Percentile_ADC’ ≥ 15.519**	Overconfidence rate	+15.11% than global	For records in the dataset where ‘log-sigma-3-0-mm-3D_firstorder_90Percentile_ADC’ ≥ 15.519, we found a significantly higher number of overconfident wrong predictions (106 samples, corresponding to 67.09% of the wrong predictions in the data slice).
**‘log-sigma-4-0-mm-3D_glszm_SmallAreaEmphasis_T2’ ≥ 0.417**	Balanced Accuracy	−13.14% than global	For records in the dataset where ‘log-sigma-4-0-mm-3D_glszm_SmallAreaEmphasis_T2’ ≥ 0.417, the balanced accuracy is 13.14% lower than the global balanced accuracy.
**‘wavelet-LLL_glcm_Imc1_DWI’ ≥ 1.615 × 10^−1^**	Precision	−7.71% than global	For records in the dataset where ‘wavelet-LLL_glcm_Imc1_DWI’ ≥ −1.615 × 10^−1^, the precision is 7.71% lower than the global precision.
**‘wavelet-LLL_glcm_Imc1_DWI’** **≤** **1.615 × 10^−1^**	Balanced Accuracy	−6.45% than global	For records in the dataset where ‘wavelet-LLL_glcm_Imc1_DWI’ < −1.615 × 10^−1^, the balanced accuracy is 6.45% lower than the global balanced accuracy.

**Table 5:** Comparison of the proposed Simplatab framework with existing AutoML frameworks on a feature-based level.

Feature	*Simplatab*	*Auto-WEKA*	*Auto-Sklearn*	*ML-Plan*	*ATM*	*Google AutoML*	*TPOT*
Optimal threshold for the models based on user needs	✓	--	--	--	--	--	--
Ease of Use	High	Moderate (setup + coding)	Moderate (coding)	Moderate (setup+coding)	Moderate (cloud + Setup+ coding)	High (cloud)	High
User-friendly interface	✓	--	--	--	--	✓	--
No code	✓	--	--	--	--	--	--
Open access	✓	✓	✓	✓	✓	--	✓
Subscription	--	--	--	--	--	✓	--
Detailed Human-readable reporting and visualization	✓	--	--	--	--	--	--
Shapley XAI analysis	✓	--	--	--	--	--	--
Data bias detection	Suite of multiple bias detection metrics	--	--	--	--	--	--
Model vulnerabilities detection	assess robustness, calibration, data leakage, performance bias, stochasticity, and confounded features	--	--	--	--	--	--
Transparency	Resulting pipelines given for all models	Best model given	Best model given	Best model given	Best model given	Resulting pipelines given for all models	Best model given
Model export	✓	--	✓	✓	✓	✓	✓
cloud integration	--	--	--	--	✓	✓	--
Community support	Open-access GitHub repository	✓	✓	✓	✓	--	✓

**Figure A1:**
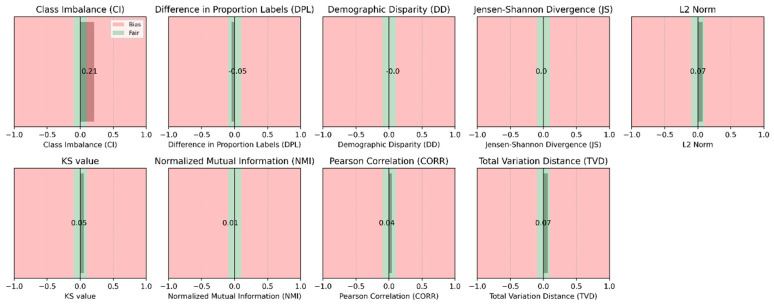
Data bias detection by client age group.

**Figure A2:**
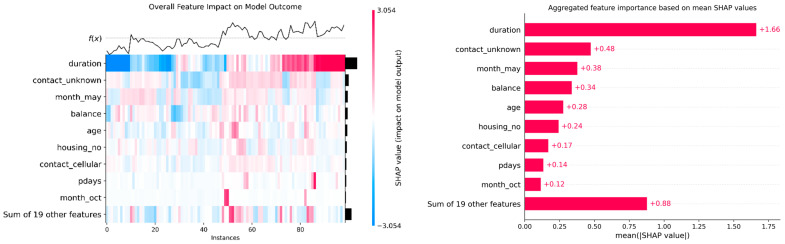
Heatmap with the SHAP values (**left**) and importance of each feature for model decision (**right**) of the XGBoost model.

**Figure A3:**
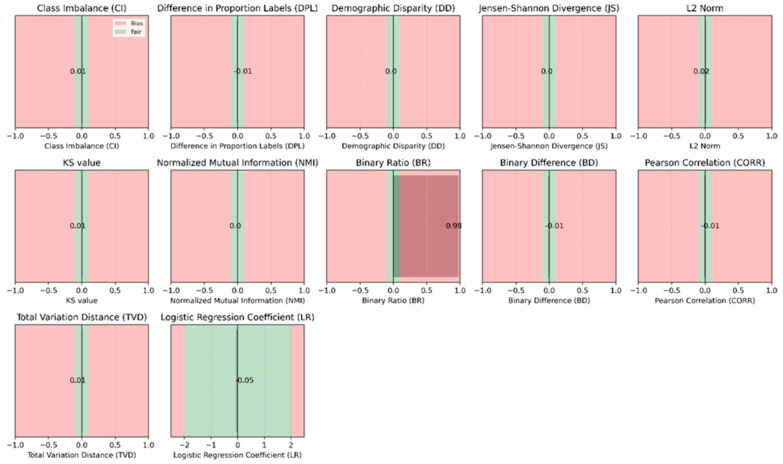
Data bias detection by customer gender (male/female).

**Figure A4:**
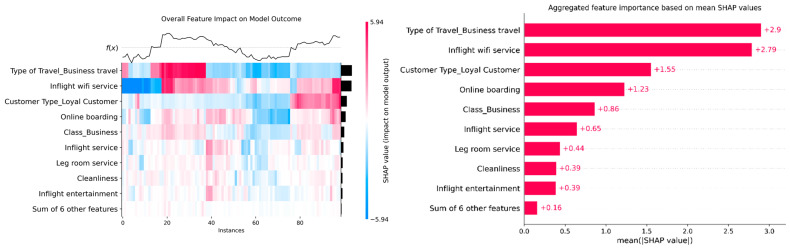
Heatmap with the SHAP values (**left**) and the importance of each feature for model decision (**right**) for the XGBoost model.

## Data Availability

The raw data supporting the conclusions of this article will be made available by the authors on request.

## Supplementary Materials

The following supporting information can be downloaded at: https://www.mdpi.com/article/10.3390/bioengineering12030242/s1. Figure S1. The Detailed Human Readable Outcomes from Simplatab Framework.

## Abbreviations

The following abbreviations are used in this manuscript:

PCa	Prostate Cancer
AI	Artificial Intelligence
ROI	Region of Interest
ML	Machine Learning
AutoML	Automated Machine Learning
TPOT	Tree-based Optimization Tool
XAI	Explainable Artificial Intelligence
ROC-AUC	Receiver Operating Characteristic—Area Under Curve
ATM	Auto Tune Models
NAS	Neural Architecture Search
PZ	Peripheral Zone
csPCa	Clinically Significant Prostate Cancer
DBD	Data Bias Detection
DB	Davies–Bouldin
CI	Class Imbalance
DPL	Difference in Proportions of Labels
DD	Demographic Disparity
JS	Jensen–Shannon
TVD	Total Variation Distance
KS	Kolmogorov–Smirnov
NMI	Normalized Mutual Information
CORR	Pearson Correlation
LR	Logistic Regression
DT	Decision Tree
MLP	Multi-Layer Perceptron
SULOV	Searching for Uncorrelated List of Variables
RFE	Recursive Feature Elimination
RF	Random Forest
SGD	Stochastic Gradient Descent
SVM	Support Vector Machines
RBF	Radial Basis Function
XGBoost	Extreme Gradient Boosting

## Appendix A

### Appendix A.1. Use Case 1: Bank Marketing Campaign Strategies

#### Appendix A.1.1. Dataset

The bank marketing dataset is used to analyze the effectiveness of telemarketing campaigns by a Portuguese banking institution. The dataset includes various demographic, socio-economic, and campaign-specific features to predict the likelihood of a client subscribing to a term deposit. Table A1 presents in detail the features of the dataset to identify whether an individual is likely to participate in the term deposit program.

**Table A1:** Bank marketing campaign strategy dataset features description.

Features	Interpretation
Age	Age of the client (groups)
Job	Type of job, e.g., ‘admin.’, ‘blue-collar’, ‘entrepreneur’, etc.
Marital	Marital status, e.g., ‘divorced’, ‘married’, ‘single’, etc.
Education	Education level, e.g., ‘basic.4y’, ‘high.school’, ‘university.degree’, etc.
Default	Has credit in default? (yes/no)
Balance	Average yearly balance in euros
Housing	Has housing loan? (yes/no)
Loan	Has personal loan? (yes/no)
Contact	Contact communication type, e.g., ‘cellular’, ‘telephone’
Day of week	Last contact day of the week
Month	Last contact month of the year, e.g., ‘jan’, ‘feb’, ‘mar’, etc.
Duration	Last contact duration in seconds
Campaign	Number of contacts performed during this campaign and for this client
pdays	Number of days that passed by after the client was last contacted from a previous campaign
Previous	Number of contacts performed before this campaign for this client
poutcome	Outcome of the previous marketing campaign, e.g., ‘failure’, ‘nonexistent’, ‘success’
y	Has the client subscribed to a term deposit? (yes/no)
Month	Last contact month of the year, e.g., ‘jan’, ‘feb’, ‘mar’, etc.
Duration	Last contact duration in seconds
Campaign	Number of contacts performed during this campaign and for this client

#### Appendix A.1.2. Results

Figure A1 presents nine bias detection metrics with respect to the age groups feature, which contains a variety of groups, and their distribution with respect to the target class. Is it indicated that the dataset is relatively fair in age distribution across classes, apart from class imbalance (CI). Especially for the DPL metric, comparing the distribution of each age group to the respective target class, it seems that no bias is evident.

Table A2 presents the results of both the internal five-fold cross-validation and the hold-out set for each supported algorithm by Simplatab. The top-performing model seems to be XGBoost, achieving a sensitivity of 90% and specificity of 79%, indicating a balanced performance.

**Table A2:** Results of five-fold cross-validation and hold-out set using six metrics.

Validation	Model	Sensitivity	Specificity	AUC	F-Score	Accuracy	Balanced Accuracy
Internal five-fold stratified CV	Decision Trees	0.45 ± 0.00	0.92 ± 0.00	0.69 ± 0.00	0.45 ± 0.00	0.87 ± 0.00	0.69 ± 0.00
	Logistic Regression	0.81 ± 0.00	0.84 ± 0.00	0.90 ± 0.00	0.54 ± 0.00	0.84 ± 0.00	0.82 ± 0.00
	Multi-Layer Neural Network	0.86 ± 0.02	0.81 ± 0.01	0.90 ± 0.00	0.52 ± 0.01	0.81 ± 0.01	0.83 ± 0.00
	Random Forest	0.86 ± 0.00	0.83 ± 0.01	0.91 ± 0.00	0.56 ± 0.01	0.84 ± 0.01	0.85 ± 0.00
	Stochastic Gradient Descent	0.77 ± 0.04	0.83 ± 0.00	0.87 ± 0.01	0.51 ± 0.01	0.82 ± 0.00	0.80 ± 0.01
	Support Vector Machines	0.78 ± 0.10	0.79 ± 0.12	0.88 ± 0.00	0.50 ± 0.09	0.79 ± 0.09	0.79 ± 0.00
	XGBoost	0.88 ± 0.00	0.80 ± 0.00	0.91 ± 0.00	0.52 ± 0.00	0.81 ± 0.00	0.84 ± 0.00
Hold-out set	Decision Trees	0.47	0.92	0.69	0.46	0.87	0.69
	Logistic Regression	0.80	0.84	0.90	0.54	0.84	0.82
	Multi-Layer Neural Network	0.91	0.78	0.91	0.51	0.79	0.84
	Random Forest	0.84	0.84	0.91	0.55	0.84	0.84
	Stochastic Gradient Descent	0.73	0.86	0.87	0.53	0.84	0.79
	Support Vector Machines	0.71	0.91	0.88	0.60	0.88	0.81
	XGBoost	0.90	0.79	0.92	0.51	0.80	0.84

Figure A2 presents the SHAP values and the important features that led XGBoost model to obtain its decision. More specifically for the heatmap plot (left), it is evident that the call duration is the most important feature for the model’s decision-making process, while the others are less important, as shown in the heatmap and the bar plot as well.

## Appendix B

### Appendix B.1. Use Case 2: Airline Customer Satisfaction

#### Appendix B.1.1. Dataset

The dataset comprises customer details from an airline, including feedback on various aspects of their flight experience and related flight data. The primary goal is to predict future customer satisfaction based on these parameters. Additionally, the dataset aims to identify which service aspects should be emphasized to increase customer satisfaction. Table A3 presents in detail the features of the dataset to identify customers’ level of satisfaction.

**Table A3:** Airline customer satisfaction features description.

Features	Interpretation
Customer ID	Unique identifier for each customer
Gender	Gender of the customer
Customer type	Whether the customer is a first-time flyer or a returning customer
Age	Age of the customer
Type of travel	Purpose of the travel, such as business or personal
Class	Travel class (economy, business, etc.)
Flight distance	Distance traveled by the customer on the flight
In-flight Wi-Fi service	Customer rating of the in-flight Wi-Fi service
Departure/arrival time convenience	Customer feedback on the convenience of departure and arrival times
Ease of online booking	Customer rating of the ease of booking their flight online
Gate location	Customer feedback on the gate location
On-board service	Feedback on the quality of on-board services
Leg room service	Customer rating of the legroom space
Check-in service	Feedback on the check-in process
Cleanliness	Customer rating of the cleanliness of the aircraft
In-flight Service	Feedback on the overall in-flight service
In-flight entertainment	Customer rating of in-flight entertainment options
Food and drink	Customer feedback on the food and beverage service
Seat comfort	Rating of the comfort of the seats
Baggage handling	Feedback on the baggage handling process
Departure delay in minutes	Duration of departure delay in minutes
Arrival delay in minutes	Duration of arrival delay in minutes
Satisfaction (target variable)	Whether the customer was satisfied or not
Baggage handling	Feedback on the baggage handling process

#### Appendix B.1.2. Results

Figure A3 presents 12 bias detection metrics with respect to gender features, containing either male or female customers. Is it indicated that the dataset is relatively fair in gender distribution across classes, apart from binary ratio (BR). Especially for the DPL metric, comparing the distribution of each gender to the respective target class, it seems that no bias is evident.

Table A4 presents the results of both the internal five-fold cross-validation and the hold-out set for each supported algorithm by Simplatab. The top-performing model seems to be XGBoost, achieving a sensitivity of 96% and specificity of 93%, indicating a balanced performance.

**Table A4:** Results of five-fold cross-validation and hold-out set using six metrics.

Validation	Model	Sensitivity	Specificity	AUC	F-Score	Accuracy	Balanced Accuracy
Internal five-fold stratified CV	Decision Trees	0.93 ± 0.00	0.93 ± 0.00	0.96 ± 0.00	0.94 ± 0.00	0.93 ± 0.00	0.93 ± 0.00
	Logistic Regression	0.90 ± 0.00	0.81 ± 0.00	0.91 ± 0.00	0.88 ± 0.00	0.86 ± 0.00	0.85 ± 0.00
	Multi-Layer Neural Network	0.95 ± 0.00	0.92 ± 0.00	0.98 ± 0.00	0.95 ± 0.00	0.94 ± 0.00	0.94 ± 0.00
	Random Forest	0.94 ± 0.00	0.93 ± 0.00	0.98 ± 0.00	0.94 ± 0.00	0.94 ± 0.00	0.93 ± 0.00
	Stochastic Gradient Descent	0.88 ± 0.01	0.82 ± 0.01	0.92 ± 0.00	0.87 ± 0.00	0.86 ± 0.00	0.85 ± 0.00
	Support Vector Machines	0.94 ± 0.00	0.92 ± 0.00	0.98 ± 0.00	0.94 ± 0.00	0.93 ± 0.00	0.93 ± 0.00
	XGBoost	0.96 ± 0.00	0.92 ± 0.00	0.98 ± 0.00	0.95 ± 0.00	0.94 ± 0.00	0.94 ± 0.00
Hold-out set	Decision Trees	0.94	0.93	0.97	0.94	0.94	0.94
	Logistic Regression	0.89	0.81	0.91	0.87	0.85	0.85
	Multi-Layer Neural Network	0.95	0.93	0.98	0.95	0.94	0.94
	Random Forest	0.94	0.93	0.98	0.94	0.94	0.94
	Stochastic Gradient Descent	0.87	0.83	0.91	0.87	0.85	0.85
	Support Vector Machines	0.94	0.93	0.98	0.94	0.94	0.93
	XGBoost	0.96	0.93	0.98	0.95	0.94	0.94

Figure A4 presents the SHAP values and the important features that led the XGBoost model to obtain its decision. More specifically for the heatmap plot (left), it is evident that the “business” type of travel and the quality of in-flight Wi-Fi service were the most important features for the model’s decision-making process, while “loyal” customers also held importance, especially for 23 samples (instances in the left figure at positions 77–100) where the SHAP values had higher magnitude.

## Appendix C

### Appendix C.1. Clinical Variables and Vendor-Specific Analysis

#### Appendix C.1.1. Results Using Retrospective and Prospective Data with Clinical Variables

The internal 10-fold cross-validation results shown in Table A5 presents significant variability in performance across the seven classifiers when using only clinical variables. Notably, SVM demonstrates high sensitivity (0.80 ± 0.39) but very low specificity (0.20 ± 0.40), potentially leading to a high false positive rate. In contrast, Logistic Regression and Multi-Layer Neural Networks yield lower sensitivity but higher specificity. Random Forest and XGBoost exhibit more balanced performance as in the radiomics features analyses, with moderate sensitivity and specificity and AUC values around 0.70. The external validation results (Table A6) show similar trends.

**Table A5:** Internal stratified 10-fold results for the retrospective dataset, solely utilizing clinical variables.

Model	Sensitivity	Specificity	AUC	F-Score	Accuracy	Balanced Accuracy
Decision Trees	0.63 ± 0.15	0.64 ± 0.15	0.69 ± 0.02	0.70 ± 0.12	0.63 ± 0.08	0.63 ± 0.02
Logistic Regression	0.44 ± 0.11	0.78 ± 0.12	0.64 ± 0.03	0.58 ± 0.07	0.53 ± 0.05	0.61 ± 0.02
Multi-Layer Neural Network	0.58 ± 0.13	0.66 ± 0.13	0.65 ± 0.01	0.67 ± 0.09	0.60 ± 0.06	0.62 ± 0.01
Random Forest	0.69 ± 0.11	0.58 ± 0.09	0.70 ± 0.03	0.75 ± 0.07	0.66 ± 0.06	0.64 ± 0.02
Stochastic Gradient Descent	0.51 ± 0.22	0.65 ± 0.20	0.63 ± 0.03	0.60 ± 0.16	0.55 ± 0.11	0.58 ± 0.02
Support Vector Machines	0.80 ± 0.39	0.20 ± 0.40	0.58 ± 0.02	0.68 ± 0.34	0.65 ± 0.19	0.50 ± 0.00
XGBoost	0.65 ± 0.17	0.61 ± 0.20	0.70 ± 0.03	0.72 ± 0.09	0.64 ± 0.08	0.63 ± 0.02

**Table A6:** External validation results for the prospective dataset, solely utilizing clinical variables.

Model	Sensitivity	Specificity	AUC	F-Score	Accuracy	Balanced Accuracy
Decision Trees	0.63	0.53	0.61	0.68	0.60	0.58
Logistic Regression	0.48	0.71	0.65	0.60	0.55	0.60
Multi-Layer Neural Network	0.51	0.60	0.59	0.60	0.54	0.55
Random Forest	0.72	0.43	0.63	0.73	0.63	0.58
Stochastic Gradient Descent	0.58	0.65	0.65	0.67	0.60	0.62
Support Vector Machines	0.56	0.66	0.58	0.63	0.61	0.58
XGBoost	0.73	0.41	0.63	0.72	0.62	0.57

#### Appendix C.1.2. Vendor-Specific Analysis

Table A7 presents the vendor-specific post hoc analysis for RF and XGBoost models using the prospective dataset. For GE scanners, both models exhibit robust performance, with AUC values of 0.74 (Random Forest) and 0.73 (XGBoost), alongside high sensitivity and F-score. In contrast, Siemens data show decreased performance, with AUC values of 0.63 and 0.64 and lower BA. Philips scanners yield superior results, with both models achieving AUC values above 0.76 and higher specificity.

**Table A7:** Vendor-specific results using the prospective dataset for Random Forest and XGBoost models.

Model	Model	Sensitivity	Specificity	AUC	F-Score	Accuracy	Balanced Accuracy
GE	Random Forest	0.78	0.60	0.74	0.80	0.73	0.69
	XGBoost	0.80	0.53	0.73	0.81	0.73	0.67
Siemens	Random Forest	0.73	0.42	0.63	0.77	0.66	0.57
	XGBoost	0.76	0.36	0.64	0.79	0.68	0.56
Philips	Random Forest	0.74	0.68	0.76	0.75	0.71	0.71
	XGBoost	0.75	0.64	0.77	0.74	0.70	0.7
