# Supplementary material for: Simplatab: An Automated Machine Learning Framework for Radiomics-Based Bi-Parametric MRI Detection of Clinically Significant Prostate Cancer
Source: Bioengineering (Basel). 2025 Feb 26;12(3):242. doi: 10.3390/bioengineering12030242 (PMC11939345; doi:10.3390/bioengineering12030242)
Supplement: Supplementary file 1 [file bioengineering-12-00242-s001.zip › bioengineering-3476769-supplementary.pdf]

bioengineering

MDPI

Supplementary Materials

Simplatab: An Automated Machine Learning Framework for Radiomics-Based Bi-Parametric MRI Detection of Clinically Significant Prostate Cancer

Dimitrios I. Zaridis [1,2,3], Vasileios C. Pezoulas [2,†], Eugenia Mylona [1,2,†], Charalampos N. Kalantzopoulos [1,2], Nikolaos S. Tachos [1,2], Nikos Tsiknakis [4], George K. Matsopoulos [3], Daniele Regge [5], Nikolaos Papanikolaou [6], Manolis Tsiknakis [7,8], Kostas Marias [7,8] and Dimitrios I. Fotiadis [1,2,*]

[1] Biomedical Research Institute, FORTH, GR 45110 Ioannina, Greece; dimizaridis@mail.ntua.gr (D.I.Z.); mylona.eugenia@gmail.com (E.M.); xkalantzopoulos@gmail.com (C.N.K.); ntachos@gmail.com (N.S.T.)
[2] Unit of Medical Technology Intelligent Information Systems, University of Ioannina, GR 45110 Ioannina, Greece; bpezoulas@gmail.com
[3] Biomedical Engineering Laboratory, School of Electrical and Computer Engineering, National Technical University of Athens, GR 15780 Athens, Greece; gmatsopoulos@biomed.ntua.gr
[4] Institute of Computer Science, FORTH, GR 70013 Heraklion, Greece; tsiknakisn@ics.forth.gr
[5] Department of Radiology, Candiolo Cancer Institute, FPO-Istituto di Ricovero e Cura a Carattere Scientifico, Strada Provinciale 142 Km 3.95, IT 10060 Candiolo, Turin, Italy; daniele.regge@ircc.it
[6] Computational Clinical Imaging Group, Champalimaud Foundation, PT 1400-038 Lisboa, Portugal; nickolas.papanikolaou@research.fchampalimaud.org
[7] Computational Biomedicine Laboratory, Institute of Computer Science, FORTH, GR 70013 Heraklion, Greece; tsiknaki@ics.forth.gr (M.T.); kmarias@ics.forth.gr (K.M.)
[8] Department of Electrical and Computer Engineering, Hellenic Mediterranean University, GR 71004 Heraklion, Greece
* Correspondence: fotiadis@uoi.gr
† These authors contributed equally to this work.

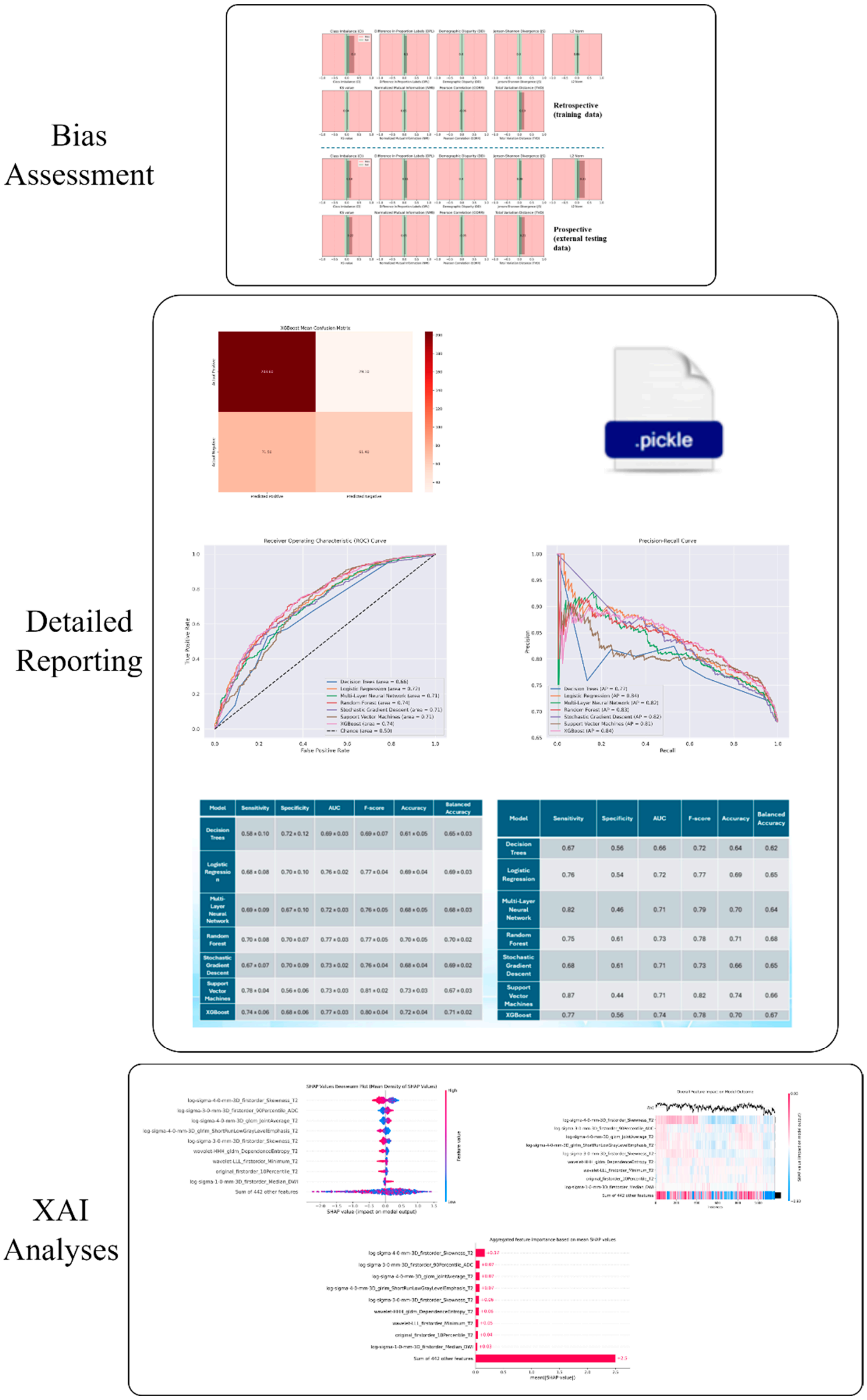

Figure S1. The Detailed Human Readable Outcomes from Simplatab Framework.

In Figure 1 is presented the overall overview of Simplatab's outcomes, including Bias assessment metrics and figures, model-related reporting in excel files and PNG images and XAI analyses utilizing Shapley values. It is important to note that the confusion matrices and the results are calculated for both the internal k-fold cross-validation and the external validation and for each algorithm the user has selected to utilize.
